# Foreign Body in the Tracheobronchial Tree as a Cause of Hemoptysis in an Adult Without Risk Factors for Aspiration: A Case Report

**DOI:** 10.7759/cureus.57596

**Published:** 2024-04-04

**Authors:** Vasiliki E Georgakopoulou, Kyriakos Tarantinos, Dimitrios Mermigkis

**Affiliations:** 1 Department of Pathophysiology and Pulmonology, Laiko General Hospital, Athens, GRC; 2 Department of Pulmonology, Sismanogleio General Hospital, Athens, GRC

**Keywords:** tracheobronchial tree, cough, hemoptysis, bronchoscopy, foreign body

## Abstract

Although aspiration of a foreign body into the trachea and bronchi can occur in all age groups, it is more common in infants and young children. Foreign bodies in the tracheobronchial tree are uncommon in adults and mainly present in patients with dysphagia and an altered level of consciousness. The identification of foreign bodies in the tracheobronchial tree is frequently overlooked or delayed, leading patients to present later with chronic symptoms and potential complications. These complications may include persistent coughing, wheezing, obstructive pneumonitis, bronchiectasis, and abscess formation secondary to recurrent pulmonary infections. This article aims to present the case of a 27-year-old patient without risk factors for aspiration who has experienced recurrent self-limiting hemoptysis episodes for five years. Bronchoscopy revealed a foreign body at the entrance to the middle lobe bronchus. The presence of a foreign body in the tracheobronchial tree should be considered in any patient with recurrent hemoptysis. Bronchoscopy leads to accurate diagnosis, treatment, and prevention of complications.

## Introduction

Hemoptysis, the expectoration of blood originating from the respiratory tract, is a concerning symptom that warrants thorough investigation and management. While it often indicates underlying pulmonary pathology such as infection, inflammation, or neoplasm, it can occasionally stem from less common etiologies [1]. Among these, foreign body aspiration into the tracheobronchial tree stands as a rare but potentially life-threatening cause, particularly in adults without predisposing risk factors [2].

Foreign body aspiration is more commonly associated with pediatric populations, often occurring during exploration or ingestion of small objects. However, its occurrence in adults, especially those without evident risk factors such as impaired consciousness, neuromuscular disorders, or altered mental status, presents a diagnostic challenge due to its rarity and the potential for delayed recognition [3].

The insidious nature of foreign body aspiration in adults, coupled with its diverse clinical manifestations, underscores the importance of considering this diagnosis even in patients lacking traditional predisposing factors. The clinical presentation of foreign body aspiration can vary widely, ranging from asymptomatic cases to severe respiratory distress or even fatal outcomes if not promptly recognized and managed [4]. Hemoptysis, although less commonly associated with foreign body aspiration compared to cough, dyspnea, or wheezing, can serve as a sentinel sign warranting further investigation [5]. The presence of hemoptysis in the absence of apparent pulmonary pathology or predisposing risk factors should prompt clinicians to maintain a high index of suspicion for foreign body aspiration, particularly in cases refractory to conventional treatment modalities [6].

Diagnostic evaluation of suspected foreign body aspiration encompasses a multifaceted approach, including detailed clinical history, physical examination, and various imaging modalities. Bronchoscopy serves as the gold standard for both diagnosis and therapeutic intervention in cases of suspected foreign body aspiration. Flexible bronchoscopy offers real-time visualization of the airways, enabling direct inspection and retrieval of foreign bodies under direct vision [7].

Management of foreign body aspiration encompasses both immediate interventions to relieve airway obstruction and subsequent measures to address associated complications, such as hemoptysis [8]. Prompt removal of the foreign body is paramount to prevent further respiratory compromise and mitigate the risk of recurrent aspiration or secondary infections [9].

Herein, we present a case report detailing the diagnostic journey and management of a foreign body lodged in the tracheobronchial tree of an adult patient, leading to hemoptysis despite the absence of typical aspiration risk factors. Through the presentation of this case report, our objective is to emphasize the diagnostic and treatment complexities linked to foreign body aspiration in adults. Additionally, we aim to underscore the necessity of maintaining a vigilant stance for this infrequent yet medically significant condition in individuals who present with unexplained hemoptysis.

## Case presentation

**Figure 1:**
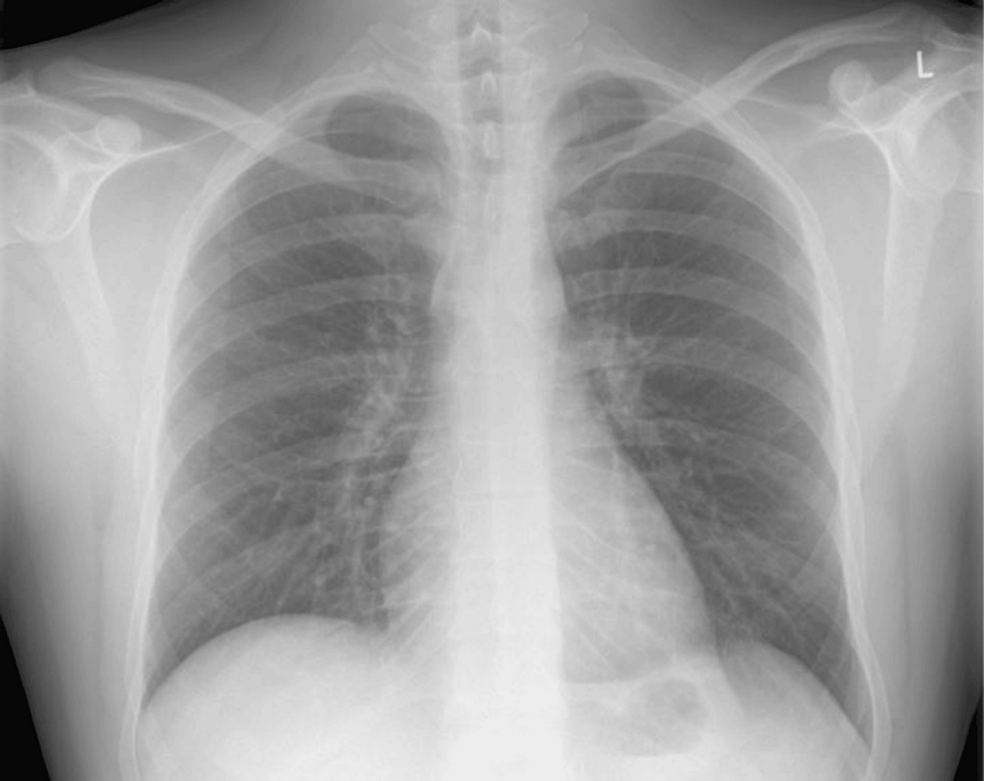
Chest X-ray

A 27-year-old non-smoking patient with a reported history of bronchial asthma treated with inhaled budesonide or formoterol presented due to recurrent self-limiting episodes of hemoptysis for five years without further investigation. The patient underwent a chest X-ray, which revealed no abnormalities (Figure 1).

**Figure 2:**
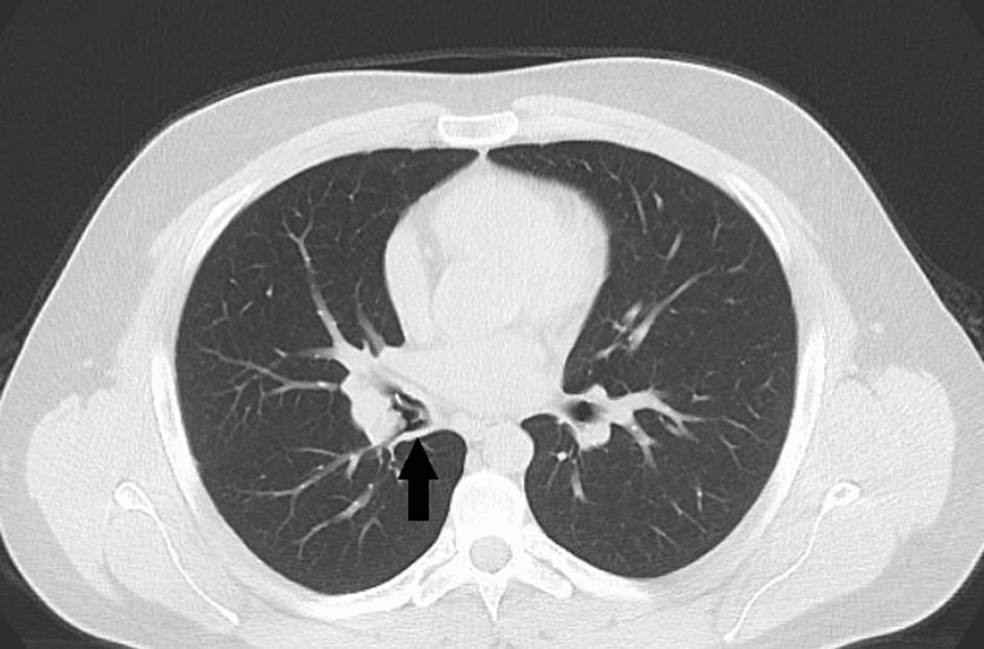
CT of the chest The arrow shows the foreign body in the right bronchus intermedius.

The patient also underwent blood clotting testing with a complete blood count and testing for prothrombin time, international normalized ratio, and partial thromboplastin time, which also did not reveal any abnormalities. Subsequently, a CT of the chest was performed, revealing the presence of a foreign body in the right bronchus intermedius (Figure 2).

**Figure 3:**
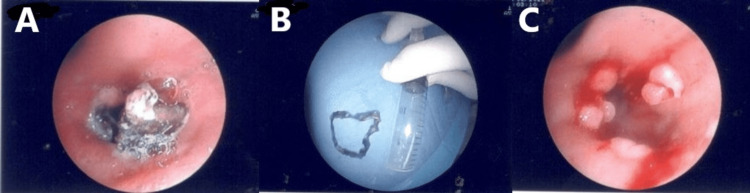
(A) Foreign body at the entrance to the middle lobe bronchus. (B) The foreign body was identified as a piece of a bottle cap. (C) After the removal of the foreign body, multiple polypoid lesions with hemorrhagic dispositions were revealed.

The patient underwent bronchoscopy, which revealed a foreign body at the entrance to the middle lobe bronchus (Figure 3A), which, after removal, proved to be a piece of plastic cap (Figure 3B). After the removal of the foreign body, multiple polypoid lesions with hemorrhagic dispositions were revealed (Figure 3C).

The patient was questioned about foreign body ingestion during the five-year period of intermittent hemoptysis, and he denied any ingestion. Follow-up evaluations at three and six months revealed no new episodes of hemoptysis.

## Discussion

Foreign body aspiration in adults often underscores its atypical presentation and diagnostic dilemma, particularly in patients lacking traditional risk factors for aspiration [4].

Despite its rarity, several case reports and case series have documented instances of foreign body aspiration presenting with hemoptysis in adults without evident predisposing factors. For instance, a case report by Narasimhan and Ashokan described a middle-aged adult presenting with hemoptysis attributed to the aspiration of a fish bone, highlighting the importance of considering foreign bodies as a differential diagnosis even in the absence of overt risk factors [10].

Similarly, Nagano et al. described a case of massive hemoptysis in an adult due to marked dilation of the inferior lobe branch of the bronchial artery caused by a foreign body. This observation underscores the diagnostic challenge posed by radiolucent or subtle foreign bodies, necessitating a high index of suspicion and the utilization of advanced imaging modalities such as CT for accurate localization and characterization [11].

Furthermore, foreign body aspiration presenting as hemoptysis in adults has been reported in association with various objects beyond food particles, including dental prostheses, metallic objects, and organic materials. In a case series by Eliçora et al. [12], hemoptysis secondary to foreign body aspiration was documented in adult patients ranging from middle-aged individuals to the elderly, highlighting the diverse demographic and clinical spectrum of this condition.

Although foreign body aspiration is a rare indication for flexible bronchoscopy in adults [13], a multicenter retrospective observational study that evaluated 138 adults for tracheobronchial foreign body aspiration revealed that foreign body aspiration is not rare, even in adults who do not have predisposing factors [14].

Diagnostic evaluation of suspected foreign body aspiration in adults often hinges on a comprehensive approach encompassing clinical history, physical examination, and radiological investigations [15]. Chest radiography remains a fundamental initial step, although its sensitivity in detecting radiolucent or subtle foreign bodies may be limited. However, as demonstrated in our case report and corroborated by existing literature, chest radiography may lack sensitivity in detecting radiolucent or small foreign bodies, necessitating adjunctive imaging modalities such as CT or bronchoscopy for a definitive diagnosis [16]. CT of the chest offers superior sensitivity and specificity, providing detailed anatomical information crucial for identifying the presence and precise localization of foreign bodies within the tracheobronchial tree [17].

Bronchoscopy, whether performed via flexible or rigid means, remains the cornerstone of both diagnosis and therapeutic intervention in cases of foreign body aspiration. Real-time visualization of the tracheobronchial tree enables direct inspection and retrieval of foreign bodies under direct vision, thereby mitigating the risk of recurrent aspirations or complications such as hemoptysis [18].

Successful extraction of the foreign body, however, may present technical challenges, particularly in cases involving complex anatomical locations or concurrent pulmonary pathology. In cases where flexible bronchoscopy fails to identify the culprit foreign body or its retrieval proves challenging, rigid bronchoscopy with its larger working channel and additional instrumentation may be required for successful extraction [19].

Management of foreign body aspiration encompasses both immediate interventions to relieve airway obstruction and subsequent measures to address associated complications, such as hemoptysis. Prompt removal of the foreign body is paramount to prevent further respiratory compromise and mitigate the risk of recurrent aspiration or secondary infections. However, the approach to foreign body removal must be tailored to the individual patient, considering factors such as the size, nature, and location of the foreign body, as well as the presence of concurrent pulmonary pathology or comorbidities [20].

## Conclusions

Our case report underscores the diagnostic and therapeutic challenges associated with foreign body aspiration-induced hemoptysis in adults without traditional risk factors for aspiration. By contextualizing our findings within the broader literature, we have elucidated common themes, diagnostic pitfalls, and management strategies relevant to this uncommon but clinically significant entity. Moving forward, continued vigilance and awareness among clinicians are essential to ensure timely recognition and appropriate management of foreign body aspiration in adults presenting with hemoptysis, thereby optimizing patient outcomes and reducing the risk of complications.
